# Dimensions of Social Stratification and Their Relation to Mortality: A Comparison Across Gender and Life Course Periods in Finland

**DOI:** 10.1007/s11205-019-02078-z

**Published:** 2019-03-18

**Authors:** Rasmus Hoffmann, Hannes Kröger, Lasse Tarkiainen, Pekka Martikainen

**Affiliations:** 1 European University Institute, Via Dei Roccettini 9, 50014 San Domenico di Fiesole, Italy; 2 Max Planck Institute for Demographic Research, Konrad-Zuse-Straße 1, 18057 Rostock, Germany; 3 German Institute for Economic Research, Mohrenstraße 58, 10117 Berlin, Germany; 4 Department of Social Research, Sociology, Center for Population, Health and Society (CPHS), University of Helsinki, PL 18, Unioninkatu 35, Helsinki, Finland

**Keywords:** Social inequality, Mortality, Health inequality, Register data, Education, Income, Occupation, Socioeconomic position

## Abstract

Differences in mortality between groups with different socioeconomic positions (SEP) are well-established, but the relative contribution of different SEP measures is unclear. This study compares the correlation between three SEP dimensions and mortality, and investigates differences between gender and age groups (35–59 vs. 60–84). We use an 11% random sample with an 80% oversample of deaths from the Finnish population with information on education, occupational class, individual income, and mortality (n = 496,658; 274,316 deaths between 1995 and 2007). We estimate bivariate and multivariate Cox proportional hazard models and population attributable fractions. The total effects of education are substantially mediated by occupation and income, and the effects of occupation is mediated by income. All dimensions have their own net effect on mortality, but income shows the steepest mortality gradient (HR 1.78, lowest vs. highest quintile). Income is more important for men and occupational class more important among elderly women. Mortality inequalities are generally smaller in older ages, but the relative importance of income increases. In health inequality studies, the use of only one SEP indicator functions well as a broad marker of SEP. However, only analyses of multiple dimensions allow insights into social mechanisms and how they differ between population subgroups.

**Electronic supplementary material:**

The online version of this article (10.1007/s11205-019-02078-z) contains supplementary material, which is available to authorized users.

## Introduction

Differences in health and mortality between groups with different socioeconomic positions (SEP) have been found in numerous studies and across gender, periods, ages, and countries (Elo 2009; Mackenbach et al. 2015). Measuring SEP is fundamental to such research; it can be influenced by data availability, cross-country comparability, or simply by the interest in a specific dimension of SEP and its relation to health. Many authors have addressed the question as to which indicators of SEP might be most appropriate for studying health inequalities (Hoffmann et al. 2019; Krieger et al. 1997; Lynch et al. 2000), and also for studying other consequences of SEP such as the educational attainment of an individual’s children (Bukodi et al. 2014). The core argument is that SEP is inherently multidimensional, and consequently should not be measured with only one indicator; furthermore, different measures should not be treated as interchangeable (Braveman et al. 2005; Geyer et al. 2006; Goldthorpe 2010), but should be studied both separately and in conjunction (Bukodi et al. 2014). Our study contributes to this discussion by empirically examining the multivariate relations and associations between mortality and the three variables education, occupational class, and income in Finland, and assessing differences by gender and age.

The concept of SEP as a multi-dimensional construct implies that all dimensions are correlated, but that each dimension has a unique relationship with health, which can be revealed by multivariate analyses, resulting in net-effects of variables that may be attributed to specific mechanisms which relate them to health. The effects of multiple SEP dimensions include the underlying temporal and causal relations between them (Galobardes et al. 2007). For example, education can have a direct effect on health, through knowledge about risk factors, risk behaviour, etc., but also an indirect one, via resources provided by occupational class and income (Lahelma et al. 2004). Thus, the net effect of a SEP variable is only part of its total effect. If SEP variables are treated as individual characteristics, it is important to “keep in mind that they are derived from larger social and economic processes that shape the distribution of education, occupation and income across the population” (Lynch and Kaplan 2000: 22). *Social* determinants of health derive their social significance and their relevance to health from social processes of attribution and distribution that take place on a non-individual level (household, neighbourhood, country), although a link to the individual level must persist to influence health.

Our study has three aims: First, we establish the relative importance of three commonly-used SEP indicators and their related mechanisms for mortality using a high-quality dataset from Finland. Second, we study how these findings differ by gender and age. Third, we illustrate the magnitude of the error if only one SEP indicator is used and all health inequalities are interpreted as the result of one dimension, if results based on different single SEP indicators are compared, or if one SEP indicator is applied to different life cycle stages and gender.

## Comparative Framework

### Pathways from Different Dimensions of SEP to Health

Education imparts knowledge regarding health risks and healthy behaviour, and provides cognitive skills like self-efficacy for dealing with complex information, such as the effect of behaviour on health and dealing with healthcare institutions. Better education promotes reduced stress, as well as better coping and preventive behaviour (Hummer et al. 1998). Research on human capital has shown that education promotes cognitive and non-cognitive skill formation. These skills in turn facilitate the accumulation of health capital through self-regulation and choices (Cunha and Heckman 2007).

The positive health effect of education as ‘learned effectiveness’ is cumulative and self-amplifying, because education increases the sense of control, which shapes healthy life styles. The perceived success in controlling one’s health determinants (e.g. weight control) creates incentives for further investments in health and other life domains (e.g. sporting activity) (Mirowsky and Ross 2003). The observed overall association between education and health can be partly explained by material factors and behaviour, because higher education means higher income and more healthy behaviour (van Oort et al. 2005). Better educated people also have more rewarding and healthier jobs, which is another indirect effect of education on health (Mirowsky and Ross 2003).

The use of education as an indicator for SEP is widespread, because of its simplicity, availability, and comparability, especially in internationally comparative studies (Eikemo et al. 2008; Mackenbach et al. 2015). However, its significance as an indicator of SEP and its benefit for health is disputed, partly due to the unclear differentiation between direct and indirect effects. Some authors argue that education is not a measure of SEP, but rather a mechanism by which individuals gain more dominant (or less dominated) positions in society, and that it has little direct effect on health (Bartley 2003; Blane 2006). Others claim that education is an important marker of SEP (Lynch and Kaplan 2000) and, if one also considers indirect effects via occupation and income, is the most important SEP dimension for health (Mirowsky and Ross 2003). Analyses of school reforms as natural experiments have demonstrated a small positive causal effect of education on health (Gathmann et al. 2015).

Occupational class influences health through the social advantages that a job can provide, and through physical and mental health risks at the workplace. In most cases these two dimensions are congruent: jobs involving substantial health risks are also those with a lower occupational class position. An important argument for occupational class as indicator for SEP is that it is a relational variable reflecting superiority, equality, and inferiority in employment conditions, which is of major importance in modern societies (Goldthorpe 2010). It is a complementary measure to the attributional variables education and income (Goldthorpe 2012). Occupation is partly determined by education and determines income, and several studies suggest that occupation does not have much effect on health net of education and income (Bassuk et al. 2002; Warren and Kuo 2003). However, there is a long research tradition demonstrating specific causal pathways from occupations with a negative effort-reward balance to stress and heart disease (Siegrist et al. 1990). A related argument is that jobs that involve more productive self-expression, rather than self-suppression, favour health, and that better educated people are more likely to find such jobs (Mirowsky and Ross 2003). The practical implementation of occupation as an SEP indicator is limited by the fact that not all people work, be they homemakers or retired people no longer exposed to current work conditions. Occupational class is thus considered less important than education and income for retired people (Hoffmann 2008; Huisman et al. 2003), but it is unknown to what extent the association between past occupational class and health decreases after retirement.

Income has been shown to be strongly associated with mortality (Martikainen et al. 2014; Tarkiainen et al. 2012). It influences health and mortality through the affordability of health care, environmental hazards, consumption (diet, housing), insecurity, and the psychological burden of being poor. Besides material explanations of the benefits of income, it also enhances effective capabilities, control, freedom, and the general ability to achieve goals (Mirowsky and Ross 2003; Robeyns 2011; Sen 1999). The effect of income on mortality has been found to be large compared to education and occupation (Duncan et al. 2002; Hoffmann 2011b). Nevertheless, there is also a strong association between education and income, partly because higher education provides better opportunities on the labour market (Autor 2014). Consequently, income (or material conditions more generally) has indeed been found to partly mediate the effect of education and occupational class (Hoffmann 2011b; Mirowsky and Ross 2003; van Oort et al. 2005). The observed association between health and income has also been partly attributed to reverse causality from health to income. The relative strength of this pathway is debated (Galama and van Kippersluis 2010; Kröger et al. 2015; Martikainen et al. 2009).

It is also argued that health inequalities that manifest via processes described above are also dependent on the distribution and investment of social and material resources at the societal level. These ‘neo-material’ factors include investments in public infrastructure such as education, health and welfare services (Lynch et al. 2000). In this regard, it is noteworthy that in international comparisons health inequalities are not systematically smaller in Finland and other Nordic welfare states than in other European regions and welfare regimes. For example, although Finland is characterized by tax-financed public provision of various social services such as child care, basic and advanced education, hospital care and health services for the elderly, average to high health inequalities are still observed (Andersen et al. 2007; Mackenbach 2012).

In our empirical analysis, the SEP-specific effects and mechanisms correspond to the net effects, i.e. controlled for the other two SEP dimensions. In this regard, the three dimensions can be compared alongside each other. Conversely, the three dimensions are deeply intertwined, complementing and operating via each other, i.e. education operating via occupation and income, and occupation operating via income. The definition of net effects and total effects depends on the variables included in a specific study. Our design can partition the effects of education into net and total effects, the latter being partly mediated by occupation and income, but we cannot separate the effects of income into net effects of material factors and behaviour.

### Gender Differences in the Determinants of Health

Health inequalities are usually larger among men than among women. This has been explained by (1) men’s greater involvement in spheres that create unequal and unhealthy living conditions, such as the working environment (Goldthorpe 1983), (2) a more unequal distribution of social resources, for example job status or income, and (3) these resources’ stronger impact on men, e.g. via unhealthy behaviour. McDonough et al. (1999) claim that the higher educational mortality gradient ensues from men receiving greater financial rewards from education. They show that the educational gradient is the same for both gender if income is controlled for.

Although mortality differentials are generally larger for men we expect this gender difference to differ between SEP variables: mortality differences between educational levels should not differ much between men and women, because the direct effects of education on health (e.g. health knowledge) should be similar across gender, as suggested by McDonough et al. (1999). The occupational gradient might be smaller for women because, as explained above, unhealthy working conditions are more common and more unequally distributed for men, or it might be smaller for men, because superiority, equality, and inferiority in employment and the effort-reward balance can be more important for women, while men define their status and success more by income. This relates to our hypothesis on the income gradient that we expect to be larger among men, because material reward and success is possibly more important for men than women, and because more women can partly rely on their partner’s contribution to the wealth of the household, which logically would result in a weaker association between women’s individual income and mortality.

### Age Differences in the Determinants of Health

We simplify the perspective on age by differentiating between working ages (35–59) and retirement ages (60–84). Most available SEP indicators are related to the labour market. Education can be understood as *input to*, occupational class as the *position in*, and income as *output of* the labour market. In modern societies, the labour market is the most important system for allocating persons to socioeconomic positions. The question arises as to whether the association between different SEP dimensions and health changes after retirement, as has been suggested in the literature (Avlund et al. 2003; Grundy and Holt 2001). This change can be motivated by a life course perspective in which education, occupational class, and material resources not only appear in a rough temporal and causal sequence across the life course, but might also have specific life course phases in which they influence health. For example, material factors may be especially important for older people, because material factors take over the role of occupational class and status after retirement (Avlund et al. 2003). We hypothesise that the educational and occupational gradients in mortality are smaller in retirement than at working age, because the direct effects of education and occupation fade over time, and that mortality differences between income groups decrease much less and thus have increasing relative importance.

When hazard ratios (HRs) for different SEP dimensions are compared between age groups, we are faced with at least two major underlying and opposing processes that hinder conclusions on the change of effects over age: First, accumulation of social and health advantages and disadvantages, which increases intra-cohort health inequalities and, second, ‘age-as-leveller’, a process which implies that health inequality decreases over time, either because of mortality selection, or because poor health and the biological processes of ageing dominate social influences (Hoffmann 2011a). The net effect seems to be that health inequality decreases at older ages, as most studies corroborate (Hoffmann 2005). We will examine this commonly found decrease for three dimensions of SEP to reveal changes in the importance of specific SEP dimensions during the life course.

## Data

Our data comes from an 11% sample from Finnish population registers aged 35 to 84 years in 1987–2007, augmented by an 80% oversampling of those who died between 1987 and 2007. The oversampling is addressed by sampling weights, reflecting unequal sampling probability. From this sample we selected those who lived in Finland in 1995, with a 13-year follow-up period until 2007. Education and occupational class are measured in the baseline year, and income is measured before the baseline year as the average of annual income data from 1987 to 1995. Further variables are age, gender, date of death, employment status, language, and region. For details about the three SEP variables, see Table 1. For details about all other variables, see Online Resource Table 1. We exclude 1.6% of the sample who have no information on occupational class, of whom 87% are retired. We further exclude 1% students, 6.7% self-employed people, and 13.2% farmers, because, first, we could not define their hierarchical occupational class position and, second, because the income data of employed and self-employed persons is not directly comparable. Online Resource Table 2 shows the remaining sample size after each of these exclusions, which result in a total sample size of 496,658 individuals with 274,316 deaths.

**Table 1:** Summary statistics for all samples

	Total sample	Women		Men	
		Age 35–59	Age 60–84	Age 35–59	Age 60–84
Education (%, baseline year 1995)					
Primary, low secondary (ISCED 0–2)	46.9	34.5	75.1	35.6	69.6
Upper secondary (ISCED 3–4)	29.4	35.9	15.1	36.4	13.3
Lowest tertiary (ISCED 5)	12.2	17.5	4.6	12.5	7.7
Higher tertiary (ISCED 6–8)	11.6	12.2	5.2	15.4	9.5
	100	100	100	100	100
Occupational class (%, in 1995)					
Non-specialized manual	22.9	18.8	33.4	20.9	24.5
Specialized manual	25.3	12.1	21.8	35.6	40.0
Lower white collar, non-managerial	13.7	26.9	11.7	5.0	3.2
Lower white collar, managerial	21.2	25.4	23.3	17.1	17.1
Upper white collar	16.8	16.8	9.7	21.4	15.3
	100	100	100	100	100
Income					
In €, average between 1987–1995	18,266	17,516	11,588	23,197	17,154
Observations	496,658	105,293	148,396	127,036	115,933
Deaths between 1995–2007 (total: 274,316)	296,793	24,022	119,363	53,289	100,119
Weighted death rate	0.170	0.039	0.361	0.090	0.465

*ISCED* International Standard Classification of Education

Percentages and death rates weighted for oversampling of deaths. For the frequencies of the income-quintiles, employment status, age, native language, region, and gender, see Online Resource Table 1

### Variables for SEP

Education was coded as four categories of highest achieved education according to the 2007 classification of Statistics Finland, which is comparable to the International Standard Classification of Education (ISCED). Occupational class is coded in five categories, according to the classification of socioeconomic groups in the Finnish registers, which is similar to the Erikson-Goldthorpe-Portocarero classification (Erikson and Goldthorpe 1992). For retired, other non-employed, and unemployed persons, this variable refers to the occupation at the last quinquennial census for which occupational data was available. For the category labels of education and occupational class, see Table 1.

Income data is derived from the registers of the Finnish tax administration and social insurance institution, defined as individual income subject to state taxation (corrected for inflation, reference year 2000). It includes wages, capital income, and taxable income transfers, for example sickness benefits, but does not include tax-free benefits, such as child benefits (not means-tested), housing allowances, and social assistance. Income quintiles are calculated from gender-specific income distributions. We used average income for 8 years before the baseline year to ameliorate the problem of reverse causation from health to income.

## Methods

A Cox proportional hazard model (Cox 1972) is used to estimate hazard ratios (HR) that show the risk of dying in a certain SEP group relative to the reference group. The process time is calendar time. Individuals who are alive at the end of the observation period or who emigrate are censored. Age at baseline (equivalent to birth cohorts) is accounted for by controlling for five-year age groups. We calculate bivariate models, and multivariate models including education, occupational class, and income. After the main models applied to the whole sample, we run separate analyses by gender and by age group (35–59 and 60–84), comparing the results along these two dimensions. The age differentiation roughly separates working ages and retirement ages. In all models we control for three potential confounders: (1) 21 regions in Finland, that mainly take into account mortality differences between southwest and northeast Finland and the regional differences in health care provision, (2) language groups that reflect the Finnish speaking majority, the Swedish speaking minority (with a higher average SEP) and other languages, and (3) employment status, which is not part of our definition of SEP but highly correlated with all SEP dimensions and mortality. Since we are interested in the gross association between SEP and mortality, we do not control for other factors such as marital status, because they are more part of the mechanisms by which SEP influences health than a confounder.

To investigate and to better compare the relative importance of the three SEP dimensions for health, we calculate the population attributable fraction (PAF), which takes the sizes of the categories of each variable into account. It expresses the total impact of a variable across its entire distribution and thus allows a better comparison of the three SEP variables. It does not assume a linear relationship between an SEP dimensions and mortality, as other commonly used summary measures for health inequality do (Relative Index of Inequality). Its calculation is based on hazard ratios for each risk category of an SEP variable, weighted by the relative size of the category (see formula below) (Miettinen 1974; Rockhill et al. 1998). It can be interpreted as the proportional reduction in overall mortality that would occur were everyone to hypothetically experience the rates of the highest socio-economic group.

Formula for the population attributable fraction

$$PAF = \sum\limits_{i = 1}^{n} {P_{i} } \left( {\frac{{HR_{i} - 1}}{{HR_{i} }}} \right)$$

n = number of exposure categories. P_i_ = proportion of population currently in the ith exposure category. HR_i_ = Hazard Ratio for mortality in the ith exposure category.

## Results

Table 1 shows the distribution of the variables in the total sample and by gender and age. Overall, the largest educational group are those with primary or low secondary education, but in the age group 35–59 the largest group are those with upper secondary education, which demonstrates the expansion of education across time and cohorts. A difference between age groups is also visible in the distribution of occupational class; higher classes are more common in the younger age group. Among men the largest group is specialized manual workers and only up to 5% are lower white collar non-managerial occupations, which represents a share of 26.9% among women aged 35–59. Among older women the largest occupational group is non-specialized manual workers. Income is higher among men and lower for older ages. In Table 2 we show the correlation between the three SEP variables, for the total sample and by gender and age. All correlations are positive, are consistently higher among men and range between 0.33 and 0.60, showing that our analysis is not limited by too high collinearity.

**Table 2:** Spearman rank correlations between the SEP-variables education, occupational class and income

	Total sample			Women						Men					
				Age 35–59			Age 60–84			Age 35–59			Age 60–84		
	Edu	Occ	Inc	Edu	Occ	Inc	Edu	Occ	Inc	Edu	Occ	Inc	Edu	Occ	Inc
Education	1.00			1.00			1.00			1.00			1.00		
Occupation	0.53	1.00		0.48	1.00		0.39	1.00		0.60	1.00		0.56	1.00	
Income	0.47	0.45	1.00	0.34	0.37	1.00	0.36	0.33	1.00	0.42	0.49	1.00	0.51	0.53	1.00

Calculated on the 11% random sample, without oversampling

Table 3 shows three bivariate models, one for each SEP variable, and then sequential models adding occupation, then income (for the HRs of all variables in the model and goodness-of-fit measures, see Online Resource Table 3). In bivariate models, each of the three SEP variables show the expected association with mortality. The hazard ratio of 1.59 for the lowest educated group in the bivariate model (and also the HRs for the other educational categories) decreases by about one half when occupation is added to the model (HR 1.31) and decreases even more when income is added (HR 1.10). This shows that most of the total effect of education on mortality occurs via occupation and income. The share of the indirect effect (IND) from the total effect can be quantified with the formula IND = (ln(1.59) − ln(1.10))/ln(1.59). This results in 79% of the difference in the mortality hazard between primary education and higher tertiary education that can be accounted for by the inclusion of education and income. The respective numbers for upper secondary and lowest tertiary education are 93% and 90%. A similar situation can be found for lower occupational classes, where the HRs decrease by about half when including income. This suggests that almost half of the negative effect of low occupational class on mortality can be explained by low income. In particular, 43% of the difference in the mortality hazard between non-specialized manual workers and upper white collar workers can be accounted for by the inclusion of income. This is not true for lower white collar non-managerial jobs, which display zero health disadvantage, and lower white collar managerial jobs, where the disadvantage is not apparently related to income.

**Table 3:** Hazard ratios for mortality from bi- and multivariate models for total sample and subpopulations

	Total sample					Women		Men	
	Income	Occupation	Education	+ Occupation	+ Income	Age 35–59	Age 60–84	Age 35–59	Age 60–84
Education									
Primary, low secondary			**1.59**	**1.31**	**1.10**	**1.25**	1.01	**1.26**	1.05
Upper secondary			**1.33**	**1.16**	1.02	1.05	**0.92**	**1.16**	1.00
Lowest tertiary			**1.11**	**1.06**	1.01	1.00	0.92	1.10	1.07
Higher tertiary			1	1	1	1	1	1	1
Occupational class									
Non-specialized manual		**1.57**		**1.34**	**1.18**	**1.20**	**1.19**	**1.31**	1.05
Specialized manual		**1.48**		**1.27**	**1.15**	**1.18**	**1.25**	**1.26**	1.00
Lower white collar, non-managerial		**1.07**		0.97	**0.96**	1.02	0.99	1.03	0.95
Lower white collar, managerial		**1.23**		**1.11**	**1.08**	1.08	**1.14**	**1.11**	0.98
Upper white collar		1		1	1	1	1	1	1
Income									
1. Quintile (poor)	**2.08**				**1.78**	**1.68**	**1.38**	**2.86**	**1.99**
2. Quintile	**1.70**				**1.47**	**1.31**	**1.25**	**1.76**	**1.54**
3. Quintile	**1.32**				**1.19**	1.02	**1.13**	**1.27**	**1.23**
4. Quintile	**1.14**				**1.07**	0.96	1.06	**1.07**	**1.13**
5. Quintile (rich)	1				1	1	1	1	1
PAF education			0.26	0.16	0.05	0.09	− 0.01	0.14	0.04
PAF occupation		0.21		0.13	0.08	0.07	0.12	0.14	0.01
PAF income	0.23				0.17	0.05	0.17	0.19	0.29

*PAF* population attributable fraction

Follow-up period: 1995–2007. Statistically significant hazard ratios (*p* < 0.05) are printed in bold. Control variables are employment status, age, native language, region, gender (except for gender-specific models). Gender- and age-specific models are multivariate models including education, occupation and income. For the coefficients of the control variables, see Online Resource Table 3

One issue that may arise with direct quantitative comparisons of the HRs between nested models is that the difference between the (log) HR is not exactly the same as the indirect effect in a linear regression model. In fact such a calculation would constitute on average a conservative estimate of the indirect effect (Breen et al. 2018). Therefore, we recalculated the indirect effects for the total sample based on an inverse probability weighting approach (IPW) that takes the difference in scale into account and allows a direct comparison (Cole and Hernán 2004; Kröger and Skopek 2017; Van der Weele 2009). This is done as a robustness check for our comparisons of total effects and net effects. The results show that 75%/84%/83% of the difference in mortality hazard between lowest tertiary/upper secondary/primary education and higher tertiary education (reference group) can be accounted for by the inclusion of occupation and income. These proportions are similar in size to the naïve results of the formula above and therefore these confirmatory analyses imply that our main analyses are not compromised by substantively relevant underestimation of indirect pathways from SEP variables to mortality.

The next question is how we can compare the relative size of the impact of the three SEP dimensions on mortality. We employ two approaches: First, a simple comparison of the HRs in Table 3 yields the tentative conclusion that mortality differences between income groups are substantially higher than between educational and occupational groups. The latter two SEP dimensions are on a similar level, taking into account the lower HRs for the lowest educated groups (1.10) applying to 46.9% of the sample, while the HRs for the lowest occupational group (1.18) apply only to 22.9%, thus to a more selected group. Second, the PAF enables a summary of the overall impact of an SEP variable across all its categories. If the uncontrolled HRs from the bivariate model for education are used for its calculation, the PAF for education is 0.26, which means that mortality could be reduced by 26% if all persons had the mortality level of the best educated. However, if the controlled HRs are used, this value is reduced to 5%, which is slightly lower than the PAF for occupation (8%) but substantially lower than the PAF for income (17%). Thus an approach that takes the sizes of the social categories into account reaches a conclusion similar to that of a simple comparison of HRs in terms of the net effects, namely that the net effect of income is higher than the net effects of education and occupation. But differently from the first approach, it suggests that the total effect of education may be higher than both the total and the net effect of income.

Finally, we are interested in differences in the relationship between education, occupation, and income between age groups and gender. Most HRs are higher among men than among women. The exception is that mortality differences between occupational classes in old age are much larger among women than among men. Table 4 shows formalized Chi square tests of gender and age differences. They reveal only weak evidence for gender differences in the educational mortality gradient, whereas mortality differences between occupational classes in old age are clearly higher among women than among men. Even larger are gender differences in the association of income with mortality. Table 4 reveals that most income categories have higher HRs for men. This pattern of gender differences is also confirmed by the PAF which is much higher among men than among women, while the PAF for occupation in old age shows the opposite.

**Table 4:** *p* values from Chi square tests for differences of hazard ratios between age and gender groups

	Test for gender differences		Test for age differences	
	Age 35–59	Age 60–84	Women	Men
Education				
Primary, low secondary	0.9766	0.4621	**0.0048** (+)	**0.0023** (+)
Upper secondary	0.1883	0.1261	0.0806	**0.0088** (+)
Lowest tertiary	0.2655	**0.0091** (+)	0.2786	0.6440
Higher tertiary				
Occupational class				
Non-specialized manual	0.2198	**0.0021** (−)	0.8820	**0.0000** (+)
Specialized manual	0.3469	**0.0000** (−)	0.3832	**0.0000** (+)
Lower white collar, non-managerial	0.8502	0.4701	0.7190	0.2630
Lower white collar, managerial	0.7343	**0.0002** (−)	0.4272	**0.0124** (+)
Upper white collar				
Income				
1. Quintile (poor)	**0.0000** (+)	**0.0000** (+)	**0.0007** (+)	**0.0000** (+)
2. Quintile	**0.0000** (+)	**0.0000** (+)	0.3335	**0.0058** (+)
3. Quintile	**0.0000** (+)	0.0538	0.0564	0.4876
4. Quintile	**0.0481** (+)	0.1650	0.0691	0.2168
5. Quintile (rich)				

*p* values below 0.05 are printed in bold and represent a test that ignores multiple testing. Following the Bonferroni-method we take multiple testing into account by dividing the normal alpha-value 0.05 by the number of tests (44), which results in a new significance threshold of *p* = 0.0011. *p* values below 0.0011 are underlined and represent a very conservative test for age and gender differences

(+) signifies that the difference is in the expected direction, i.e. social mortality differences are smaller among women and in old age; (−) signifies an opposite finding

In the age group 60–84 almost all HRs are smaller than in the age group 35–59. In particular, education and (male) occupational class seem to have almost no net effect on mortality in old age. The exception of high female mortality differences between occupational groups in old age was already mentioned above. The tests in Table 4 confirm that HRs tend to be smaller among older people, and shows that age differences are more pronounced among men. There are no clear differences between the three SEP dimensions with regard to changes over age (except for occupational class among women). In contrast, the PAF results suggest that the overall impact of income increases over age: in old age, 29% of all deaths among men and 17% of deaths among women are attributable to income differences. Especially among men, income seems to be even more dominant over the other SEP dimension than in young ages. The PAF results can be explained by the fact that (1) the HRs for the 3rd and 4th income quintile are not much smaller, or even higher, in older ages and (2) in the age group 60–84 there are more people in the low income quintiles than in the age group 35–59 (see Online Resource Table 1). The median income quintile in the age group 35–59 is the 4th quintile, while in the age group 60–84 it is the 2nd quintile. The PAF takes these distributional differences between the age groups into account and shows the growing importance of income with age.

## Discussion

Our comparative study on the relationship between dimensions of SEP and mortality shows, first, that the association between education and mortality is substantially reduced by taking occupation and income into account. Likewise, the association between occupation and mortality is substantially reduced by taking income into account. Second, looking at the remaining net effects, the effect of income is much larger than the effect of education and occupation. Third, there is a general decrease in social mortality gradients in old age, with the exception of occupational class among women. However, taking the overall impact of the variables across their entire distribution into account, the importance of income seems to increase with age. Among men in old age, income seems to be the only SEP dimension that creates a social mortality gradient. Fourth, health inequalities are generally larger among men than among women, again with the exception of occupational class in old age, which is more important among women, while income is much more important for men.

The first finding directly relates to life course influences. The effect of education is to a large extent mediated through occupation and income, important aspects of SEP that develop during the life course after and as a consequence of education. Likewise, the effect of occupational class on mortality works to a large extent through income. The total effect of education (direct and indirect effects) is large, so the interpretation from the life course perspective is not to underestimate the importance of education as the beginning of a process in which SEP develops through several important steps, but to be in a better position to assess the relative contribution of each SEP dimension in terms of their specific mechanisms. Our second finding, that income has the largest net effect, suggests that it is generally more important what money can buy—effectiveness and freedom, including the psychological reward of being well-off as a marker for success—than knowledge, skills, and ‘learned effectiveness’. However, these estimates refer to the direct effects on health and it is important to acknowledge that education is normally a prerequisite for higher income. Beyond the processual complexity of education, occupation, and mortality in the life course, there may also be interactions between these factors that we could not take into account. For example, high education may reduce and compensate for low income because it increases the sense of control and both resources can foster effectiveness. If such interactions exist, our standard PAF method can overestimate the impact of SEP dimensions, even when adjusted hazard ratios are used for the PAF calculation (Bruzzi et al. 1985). This has been illustrated in a study comparing standard PAF calculations to more ‘direct’ approaches (‘sequential and average attributable fractions’) (Azimi et al. 2014). The latter approaches simulate different interventions, i.e. removal of risk factors, in different sequences and obtain average PAF from these simulations (Eide and Gefeller 1995). We think that our standard PAF calculations using unadjusted as well as adjusted hazard ratios are sufficient to complement the results from the Cox regression and to take into account the relative size of the different exposure categories. However, more research is needed to fully account for different interactions between SEP measures in simulation based counterfactual frameworks.

Our findings on the interactions of the SEP dimensions with gender show that men do not have higher gradients in all SEP dimensions. As hypothesized, the net effect of education does not differ substantially between gender, probably because the direct effects of education on health are similar, as suggested by McDonough et al. (1999). Furthermore, the contribution of both genders to workforce in Finland is relatively equal. During the study period the contribution of women to total paid working hours of the work-force was only around 4 percentage points lower than the contribution of men (Haataja 2006). Occupational class in old age is even more influential among women than men, i.e. for women it does not lose its importance with increasing age. This speaks against an explanation based on unhealthy working conditions. It rather suggests that superiority, equality, and inferiority in employment and the effort-reward balance are at least as important for women as for men at young ages, and become especially important for women after retirement as a source of self-assurance and social status that may also manifest through social networks at work that are maintained after retirement. However, it is also possible that this is a cohort effect among women whose manual work in the past had an especially high impact on health. Finally, as expected, income matters more for men than for women. At least in old age, this goes hand in hand with the lower impact of occupational class among men. The larger relative importance of income for men, which implies smaller direct effects of education and occupation, may be due to three factors: First, income as a marker for SEP might be more important among men because (internalized) social norms expect men to be more stable financially than women, which in turn is related to the male breadwinner model and disadvantages for women on the labour market. Second, the family ameliorates the detrimental effects of poverty. Couples can rely on their partners’ income (Torssander and Erikson 2009), and in many families, men still earn more and have more responsibility for the household income. This also implies that personal income is a less accurate measure of material conditions for women and therefore the disparity in mortality is smaller. Future research should investigate how gender differences in the impact of SEP-variables on mortality depend on marital status, because there is conflicting evidence: Koskinen and Martelin (1994) found that smaller social mortality gradients among women only exist among married people, while Montez et al. (2009) found that among married people the educational mortality gradient is the same for men and women. Third, in poverty, men are more likely to engage in health-threatening behaviour than women. More detailed studies from Finland have shown the increasing contribution of alcohol and smoking to health inequalities by income and the interplay between alcohol, poverty, and other social variables (Martikainen et al. 2014; Tarkiainen et al. 2016).

Regarding age differences, we hypothesized that mortality gradients between educational and occupational groups decline with age, while the income mortality gradient declines less strongly, leading to the higher relative importance of income. The HRs and Chi square tests confirm a decreasing importance of several risk categories of all SEP dimensions, again, except for occupational class among women. The general decrease over age may be partly attributable to the fact that most high risk groups assume a larger share of the population in old age than in young ages, which makes them less selective and more normal. But also in cases where this is not the case (e.g. upper secondary education) we see a strong decline of the mortality gradient. Taking into account distributional changes by the PAF reveals an increasing overall impact of income with age. It is noteworthy that while the PAF takes most distributional differences into account and improves the comparison of age groups, it still depends on the size of the reference group, to which all groups adapt in the scenario. To further investigate how distributional changes influence our conclusions on the impact of variables over age, we performed a sensitivity analysis based on age-specific income quintiles. This produces similar income distributions in both age groups. The results are shown in the Online Resource Table 4 and reveal that this change slightly reduces the income mortality gradients in younger ages, which logically reduces the decrease of the gradients over age, but the overall findings do not change.

Our life course framework can explain this from two perspectives: direct effects of education and occupation lose impact after retirement, because the time for long-term investment in the labour market and occupational exposure is over and what matters is the material outcome of these processes. The effect of education as the start of an SEP trajectory is transferred to occupation in mid-life and to income in older ages, when life time earnings, savings, and capital accumulate. This means that in old age the effects of education and occupation are not direct but indirect (McGovern and Nazroo 2015). This contradicts the view that education is an important SEP dimension after retirement, determining peoples’ lifestyle and the way they deal with aging when the impact of employment lessens (Ross and Wu 1996).

We acknowledge the limitations of our comparative approach in addressing fundamental and complex causal issues related to measuring health inequalities over the life course. Differences in health inequalities between age groups are the result of many factors and not only reflect the changing effect of an SEP dimension, but include processes such as accumulation, ‘age-as-leveler’, and selection which may make the age group 60–84, survivors from the age group 35–59, more homogeneous (Hoffmann 2011a). Differences over age can also result from cohort effects; for example, the decreasing mortality gradient between educational groups may be due to the expansion of education, meaning that in later cohorts, less educated individuals form a smaller, more selected group, while they represented the majority some decades ago.

Further limitations include that we cannot be sure that associations between SEP and mortality reflect causality. For example, critics of income as an SEP measure claim that the high HRs between income groups are upward-biased by reverse causation from health to income. To address this bias, we measured average income over eight years before the baseline, but to study the magnitude of reverse causality goes beyond the scope of this study (Hoffmann et al. 2018). This uncertainty about causality also affects the interpretation of the PAF results. We do not claim that the percentages of mortality reduction describe realistic what-if-scenarios, because such conclusions would assume causal effects of the risk factors (Hoffmann et al. 2013). Rather we use the PAF as a summary measure of associations across several risk categories to take into account the effect of distributional differences between variables, age groups, or gender.

Since the interplay between SEP dimensions are highly context specific, we do not know the extent to which our results are generalizable to other countries. A similar study for Sweden found similar results when several SEP dimensions were compared between gender for the age group 35–59 (Torssander and Erikson 2010). This relative similarity between Finland and Sweden is confirmed by other studies comparing social mortality gradients between Scandinavian and European countries (Mackenbach et al. 2016; Mortensen et al. 2016). This would imply that the main associations observed in this study are generalizable at least to countries with a relatively generous welfare provision system.

Finally, our aim was to reflect on the use of SEP variables in the study of health inequalities. Our results illustrate that total effects based on models with one single SEP dimension differ substantially from net effects. Only the latter can be used for investigating the individual contribution of each SEP dimension. Using several SEP dimensions allows us to separate total and net effects which, combined with theoretical background knowledge, enables insights on the mediation between the effects of SEP dimensions and between SEP and health.

Overall, our study showed that, in most subgroups, income produced the steepest social mortality gradient, but its relative importance and the role of education and occupational class varies by gender and age. Thus, for the important goal of comparing health inequalities between different settings and subpopulations, a multivariate approach is important. While it might seem ideal to always measure SEP with at least three different variables, this will not be possible in most studies, and may not even be necessary. For a descriptive or a simple comparative purpose, one indicator (e.g. income for men) might be sufficient, because it can capture important aspects of SEP and their relation to health even when other factors are not controlled for. In such a case, it would function as a marker for SEP and not for a specific health-related resource or mechanism. However, if the intention is to explain health inequalities and reveal mechanisms, or to compare gender or age groups, is important to use a multi-dimensional approach and disentangle the group-specific complexity behind health inequalities.

## Electronic supplementary material

Below is the link to the electronic supplementary material.

Supplementary material 1 (PDF 392 kb)
