# Supplementary material for: Dimensions of Social Stratification and Their Relation to Mortality: A Comparison Across Gender and Life Course Periods in Finland
Source: Soc Indic Res. 2019 Mar 18;145(1):349–65. doi: 10.1007/s11205-019-02078-z (PMC6620240; doi:10.1007/s11205-019-02078-z)
Supplement: Supplementary file 1 — Supplementary material 1 (PDF 392 kb) [file 11205_2019_2078_MOESM1_ESM.pdf]

Electronic Supplementary Material

Online Resource Table 1: Frequencies of all variables in all samples

		All	Women, 35-59	Women, 60-84	Men, 35-59	Men, 60-84
**EDUCATION**	Primary, low secondary (ISCED 0-2)	46.9	34.5	75.1	35.6	69.6
	Upper secondary (ISCED 3-4)	29.4	35.9	15.1	36.4	13.3
	Lowest tertiary (ISCED 5)	12.2	17.5	4.6	12.5	7.7
	Higher tertiary (ISCED 6-8)	11.6	12.2	5.2	15.4	9.5
**OCCUPATIONAL CLASS**	Non-specialized manual	22.9	18.8	33.4	20.9	24.5
	Specialized manual	25.3	12.1	21.8	35.6	40.0
	Lower white collar, non-managerial	13.7	26.9	11.7	5.0	3.2
	Lower white collar, managerial	21.2	25.4	23.3	17.1	17.1
	Upper white collar	16.8	16.8	9.7	21.4	15.3
**INCOME**	Avg. income 1987-1995 (in € from the year 2000)	18,266	17,516	11,588	23,197	17,154
	1.Quintil (max 7216 for women, 105690 for men)	14.6	8.0	26.4	10.1	27.6
	2.Quintil (max 11439, 16734)	18.8	11.0	34.0	14.3	30.3
	3.Quintil (max 15883, 21913)	21.4	22.7	18.7	22.7	18.5
	4.Quintil (max 20326, 28711)	22.6	28.9	11.2	26.4	11.4
	5.Quintil	22.6	29.5	9.7	26.4	12.2
**EMPLOYMENT STATUS**	Employed	50.1	72.6	4.0	68.9	5.2
	Unemployed	12.9	14.7	4.8	17.9	6.7
	Non-employed	2.7	4.6	0.8	2.7	0.3
	Pensioners	34.2	8.1	90.5	10.4	87.9
**GENDER**	Women	54.0	100	100	0	0
	Men	46.0	0	0	100	100
**NATIVE LANGUAGE**	Finnish	93.4	93.9	92.5	93.9	92.3
	Swedish	5.8	5.3	7.3	5.0	7.5
	Other	0.7	0.8	0.2	1.1	0.3
**REGION**	Metropolitan area	18.6	20.0	17.5	18.7	16.0
	Other Uusimaa	6.7	7.1	5.4	7.2	5.9
	Varsinais-Suomi	8.6	8.5	9.3	8.2	9.1
	Satakunta	4.8	4.6	5.1	4.6	5.2
	Kanta-Häme	3.3	3.1	3.7	3.3	3.5
	Pirkanmaa	9.0	8.8	9.9	8.6	9.5
	Päijät-Häme	4.1	4.1	4.4	4.0	4.0
	Kymenlaakso	4.2	3.7	4.5	4.2	4.7
	Etelä-Karjala	3.0	2.7	3.3	3.0	3.1
	Etelä-Savo	3.3	3.1	3.8	3.2	3.7
	Pohjois-Savo	4.8	4.6	4.9	4.9	4.8
	Pohjois-Karjala	3.3	3.2	3.3	3.4	3.4
	Keski-Suomi	5.1	5.0	4.9	5.1	5.5
	Etelä-Pohjanmaa	3.3	3.3	3.4	3.2	3.4
	Pohjanmaa	3.1	3.1	3.4	2.9	3.4
	Keski-Pohjanmaa	1.2	1.2	1.1	1.2	1.2
	Pohjois-Pohjanmaa	6.0	6.3	5.2	6.4	5.6
	Kainuu	1.8	1.7	1.6	1.9	2.0
	Lappi	3.8	3.7	3.3	4.1	4.0
	Itä-Uusimaa	1.7	1.7	1.6	1.7	1.7
	Ahvenanmaa	0.5	0.5	0.4	0.5	0.5
**AGE**	35-39	14.7	21.1	0	21.7	0
	40-44	15.5	22.3	0	22.6	0
	45-49	16.5	23.8	0	24.1	0
	50-54	12.1	17.8	0	17.4	0
	55-59	10.1	15.1	0	14.2	0
	60-64	8.8	0	25.4	0	32.6
	65-69	8.3	0	25.2	0	28.4
	70-74	6.8	0	22.5	0	20.6
	75-79	4.4	0	15.8	0	11.6
	80-84	2.9	0	11.1	0	6.8

Weighted to account for oversampling of deaths.

Online Resource Table 2: Sample sizes with and without four sample restrictions, by gender and age

	Full sample	Consecutively excluding… Unknown Occup. Class	Students	Self-employed	Farmers
Women, 35-59	123,360	122,253	119,206	111,772	105,293
Women, 60-84	199,674	194,489	194,041	185,057	148,396
Men, 35-59	157,029	155,624	152,713	137,584	127,036
Men, 60-84	161,357	158,838	158,630	147,133	115,933
Total	641,420	631,204	624,590	581,546	496,658

Online Resource Table 3: Hazard ratios from all variables from all models

						Women		Men	
	Income	Occupation	Education	+Occupation	+Income	35-59	60-84	35-59	60-84
Primary, low secondary (ISCED 0-2)			**1.586**	**1.308**	**1.099**	**1.253**	1.012	**1.256**	1.051
Upper secondary (ISCED 3-4)			**1.334**	**1.156**	1.019	1.046	**0.915**	**1.161**	0.995
Lowest tertiary (ISCED 5)			**1.114**	**1.059**	1.004	1.000	0.915	1.098	1.068
Higher tertiary (ISCED 6-8)			1	1	1	1	1	1	1
Non-specialized manual		**1.565**		**1.339**	**1.177**	**1.203**	**1.192**	**1.312**	1.051
Specialized manual		**1.475**		**1.267**	**1.146**	**1.177**	**1.247**	**1.259**	1.001
Lower white collar, non-managerial		**1.066**		0.967	**0.955**	1.017	0.993	1.032	0.954
Lower white collar, managerial		**1.233**		**1.109**	**1.081**	1.083	**1.138**	**1.109**	0.981
Upper white collar		1		1	1	1	1	1	1
1.Quintil (poor)	**2.081**				**1.783**	**1.678**	**1.375**	**2.857**	**1.985**
2.Quintil	**1.695**				**1.469**	**1.318**	**1.248**	**1.764**	**1.542**
3.Quintil	**1.318**				**1.186**	1.017	**1.125**	**1.270**	**1.229**
4.Quintil	**1.142**				**1.065**	0.963	1.059	**1.067**	**1.131**
5.Quintil (rich)	1				1	1	1	1	1
Employed	1	1	1	1	1	1	1	1	1
Unemployed	**1.949**	**2.306**	**2.327**	**2.272**	**1.966**	**1.646**	0.967	**1.791**	**1.286**
Non-employed	**1.608**	**2.385**	**2.331**	**2.339**	**1.707**	**1.323**	**1.297**	**1.661**	1.160
Pensioners	**2.951**	**3.509**	**3.529**	**3.453**	**2.972**	**3.561**	**1.559**	**2.644**	**1.690**
Women	1	1	1	1	1				
Men	**1.868**	**1.842**	**1.915**	**1.865**	**1.849**				
Finnish	1	1	1	1	1	1	1	1	1
Swedish	**0.942**	**0.960**	**0.951**	**0.966**	**0.958**	0.944	0.967	**0.853**	0.986
Other	**0.518**	**0.625**	**0.590**	**0.610**	**0.536**	**0.456**	**0.739**	**0.319**	0.774
Metropolitan area	1	1	1	1	1	1	1	1	1
Other Uusimaa	**0.905**	**0.925**	**0.938**	**0.916**	**0.889**	**0.814**	**0.976**	**0.857**	**0.899**
Varsinais-Suomi	**0.855**	**0.895**	**0.910**	**0.888**	**0.843**	**0.786**	**0.888**	**0.831**	**0.870**
Satakunta	**0.819**	**0.870**	**0.893**	**0.865**	**0.807**	**0.790**	**0.876**	**0.827**	**0.802**
Kanta-Häme	**0.872**	**0.911**	**0.933**	**0.907**	**0.861**	**0.803**	**0.924**	**0.853**	**0.863**
Pirkanmaa	**0.847**	**0.887**	**0.910**	**0.885**	**0.838**	**0.717**	**0.895**	**0.823**	**0.861**
Päijät-Häme	**0.879**	**0.929**	**0.950**	**0.925**	**0.869**	**0.801**	**0.920**	**0.862**	**0.879**
Kymenlaakso	**0.944**	0.977	1.005	0.975	**0.931**	**0.856**	0.970	1.006	0.953
Etelä-Karjala	**0.903**	**0.942**	0.960	**0.935**	**0.888**	**0.838**	**0.876**	0.944	0.954
Etelä-Savo	**0.867**	**0.932**	**0.950**	**0.925**	**0.858**	**0.772**	**0.919**	**0.796**	**0.879**
Pohjois-Savo	**0.870**	**0.931**	**0.954**	**0.927**	**0.861**	**0.724**	0.958	**0.773**	**0.881**
Pohjois-Karjala	**0.835**	**0.899**	**0.922**	**0.894**	**0.825**	**0.750**	**0.885**	**0.753**	**0.868**
Keski-Suomi	**0.842**	**0.897**	**0.918**	**0.892**	**0.832**	**0.687**	0.951	**0.776**	**0.827**
Etelä-Pohjanmaa	**0.795**	**0.862**	**0.880**	**0.855**	**0.785**	**0.665**	**0.868**	**0.691**	**0.829**
Pohjanmaa	**0.756**	**0.799**	**0.815**	**0.791**	**0.744**	**0.692**	**0.820**	**0.724**	**0.742**
Keski-Pohjanmaa	**0.742**	**0.795**	**0.806**	**0.785**	**0.731**	**0.603**	**0.838**	**0.671**	**0.743**
Pohjois-Pohjanmaa	**0.841**	**0.888**	**0.906**	**0.882**	**0.831**	**0.672**	**0.936**	**0.740**	**0.876**
Kainuu	**0.847**	**0.903**	**0.928**	**0.897**	**0.834**	**0.696**	0.924	**0.789**	**0.857**
Lappi	**0.891**	**0.930**	**0.955**	**0.926**	**0.877**	**0.745**	1.002	**0.745**	**0.920**
Itä-Uusimaa	**0.935**	0.972	0.988	0.961	**0.916**	0.873	0.966	0.928	0.913
Ahvenanmaa	0.913	0.906	0.917	0.899	**0.895**	0.817	0.955	0.918	0.882
35-39	1	1	1	1	1	1		1	
40-44	**1.545**	**1.511**	**1.494**	**1.498**	**1.525**	**1.496**		**1.518**	
45-49	**2.247**	**2.147**	**2.080**	**2.098**	**2.188**	**2.044**		**2.213**	
50-54	**2.999**	**2.792**	**2.668**	**2.706**	**2.892**	**2.627**		**2.992**	
55-59	**3.313**	**2.977**	**2.795**	**2.852**	**3.157**	**2.714**		**3.342**	
60-64	**3.711**	**3.371**	**3.135**	**3.212**	**3.538**		**0.100**		**0.183**
65-69	**4.946**	**4.863**	**4.501**	**4.605**	**4.750**		**0.152**		**0.254**
70-74	**8.084**	**8.474**	**7.831**	**8.002**	**7.804**		**0.288**		**0.376**
75-79	**14.38**	**15.20**	**14.12**	**14.37**	**13.85**		**0.539**		**0.615**
80-84	**25.81**	**27.33**	**25.51**	**25.82**	**24.71**		1		1
Pseudo R2	0.063	0.062	0.081	0.082	0.083	0.028	0.051	0.037	0.038
AUROC	0.818	0.817	0.816	0.817	0.819	0.701	0.726	0.736	0.676

AUROC = Area Under the Receiver Operating Characteristic. In addition to the commonly used Pseudo R2 which does not have a clear interpretation in survival models, we evaluate the overall model fit by a classification measure, namely the area under the curve based on the receiver operating characteristic (AUROC) developed for survival analytic models (Heagerty and Zheng 2005). The AUROC is a measure of how well the model can distinguish between those who die and those who do not die based on the model predictions. A value of 0.5 means no discriminatory power of the model, while 1 would mean perfect prediction. The number can be interpreted as the probability that a randomly chosen pair of persons, one who died and one who did not die, are ranked by the model prediction in such a way that the person of the pair who has a higher probability of dying (based on the model prediction) actually died (Heagerty, P.J., Zheng, Y. (2005). Survival Model Predictive Accuracy and ROC Curves, *Biometrics* 61(1):92–105).

Online Resource Table 4: Hazard ratios for mortality from multivariate models for subpopulations (gender- and age-specific income quintiles)

	Women		Men	
	Age 35-59	Age 60-84	Age 35-59	Age 60-84
**EDUCATION**				
Primary, low secondary	**1.27**	1.03	**1.25**	**1.09**
Upper secondary	1.06	0.93	**1.16**	1.03
Lowest tertiary	1.01	0.93	1.09	**1.10**
Higher tertiary	1	1	1	1
**OCCUPATIONAL CLASS**				
Non-specialized manual	**1.21**	**1.20**	**1.31**	1.05
Specialized manual	**1.18**	**1.26**	**1.25**	1.02
Lower white collar, non-managerial	1.02	1.00	1.02	0.96
Lower white collar, managerial	1.08	**1.15**	**1.10**	1.00
Upper white collar	1	1	1	1
**INCOME**				
1. Quintile (poor)	**1.48**	**1.34**	**2.59**	**2.04**
2. Quintile	**1.03**	**1.27**	**1.48**	**1.64**
3. Quintile	0.94	**1.19**	**1.18**	**1.42**
4. Quintile	0.93	**1.12**	**1.09**	**1.17**
5. Quintile (rich)	1	1	1	1
PAF Education	0.10	0.01	0.13	0.07
PAF Occupation	0.08	0.13	0.14	0.02
PAF Income	0.03	0.13	0.21	0.23

PAF = Population Attributable Fraction. Follow-up period: 1995-2007. Statistically significant hazard ratios ($p<0.05$) are printed in bold. All models are multivariate models including education, occupation and income. Control variables are employment status, age, native language, and region.
